# Impact of Resident Post-Graduate Year on Laparoscopic Cholecystectomy Outcomes

**DOI:** 10.7759/cureus.36644

**Published:** 2023-03-24

**Authors:** Mehdi Bourakkadi Idrissi, Hicham El Bouhaddouti, Ouadii Mouaqit, Abdelmalek Ousadden, El Bachir Benjelloun

**Affiliations:** 1 Department of Visceral Surgery, Hassan II University Hospital, Fez, MAR

**Keywords:** learning curve, patients' outcomes, complications, residents in training, laparoscopic cholecystectomy

## Abstract

Introduction

Laparoscopic cholecystectomy is a minimal access procedure in which the gallbladder is removed by laparoscopic techniques. Effective training for laparoscopic surgery should focus on not only understanding the anatomy and procedural steps but also acquiring the specific gestures and techniques of this type of surgery that may differ from those used in traditional open surgery. The aim of our study was to analyze whether the laparoscopic cholecystectomy performed by surgeons in training is a safe procedure.

Material and methods

This is a retrospective review of 433 patients who were divided into two groups: laparoscopic cholecystectomies performed by trainees and those performed by senior surgeons.

Results

Around 66% of surgeries were performed by resident surgeons. There was no demographic difference between residents and senior surgeons. Operative time was significantly longer in the residents’ group compared to senior surgeons’ group (96 minutes vs 61 minutes; p<0.001). The overall intra- and post-operative complication rates were 3.1% and 2.5%, respectively, with no significant difference between the two groups (p=0.368 and p=0.223). Conversion to open laparotomy was required in 8% of cases in each group (p=0.538). The mean length of hospital stay after surgery was significantly longer in patients operated by residents (p<0.001). We did not notice any case of mortality in both groups.

## Introduction

Laparoscopic cholecystectomy (LC) is a minimally invasive procedure that involves the removal of the gallbladder using laparoscopic techniques. Laparoscopy offers the significant benefit of enabling the entire surgical team to view the surgical field from the surgeon's perspective. This is a valuable educational experience for trainee surgeons as opposed to open surgery, where their vision may be restricted during certain stages of the procedure. Nevertheless, laparoscopic surgery is not without its constraints, which include the deficiency of tactile sensation, a two-dimensional visual field, inadequate instrument maneuverability, and the non-existence of inherent hand-eye coordination [1].

To adequately prepare for laparoscopic surgery, it is crucial to not solely concentrate on comprehending the anatomy and the sequential steps of the procedure; it is important to acquire the distinct movements and techniques required for laparoscopic surgery, which may vary from those used in conventional open surgery [2].

Surgical residents in Morocco have always received training in cholecystectomy as an integral part of their program. However, since the introduction of LC at our institution in the early 2000s, it has quickly become the preferred procedure for treating gallstones. This has resulted in a growing number of LCs being performed by supervised residents.

Our study aimed to determine the safety of LC performed by surgeons in training by comparing their perioperative and post-operative complications, length of hospital stay, morbidity, and mortality to those of staff surgeons who performed the same procedure. The secondary objective was to examine potential variances among the cohort of trainee surgeons as their learning curve progressed.

## Materials and methods

Through a cross-sectional retrospective study, we reviewed the medical records of all patients who underwent LC from January 1, 2017, to December 31, 2020, at the Department of Visceral Surgery at the Hassan II University Hospital of Fez, Morocco. Operative and clinical notes of all patients who underwent LC were reviewed retrospectively and the relevant data were collected. Patients were divided into two groups: LCs performed by senior surgeons versus LCs performed by trainees. The subgroup of residents was also studied in order to assess their learning curve.

**Figure 1:**
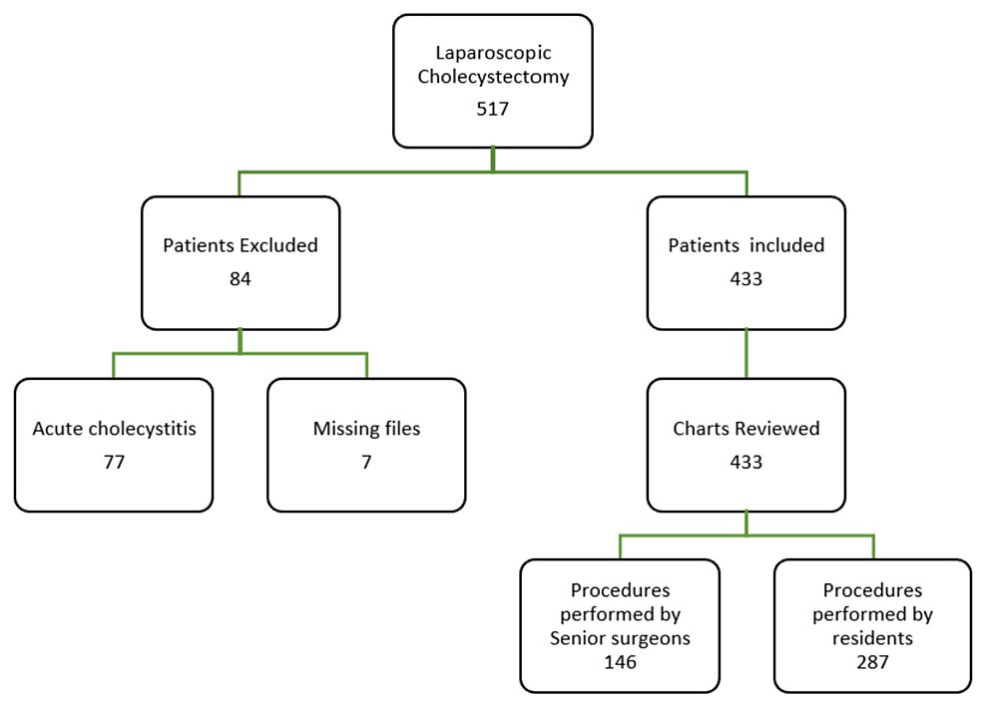
Patients selection

All patients older than 18 years of age, who underwent an LC for uncomplicated gallstone disease, were included. The exclusion criteria involved patients with complicated gallstone disease (acute cholecystitis, cholecystoenteric fistula, and gallbladder neoplasm) (Figure 1). All patients were included in the American Society of Anesthesiologists (ASA) grade 1 or 2 and underwent preoperatively abdominal ultrasound, chest X-ray, electrocardiography, and routine blood tests including liver function tests.

Nine senior surgeons with more than 15 years of experience in laparoscopic surgery and 19 resident trainees were included in the study. In our institution, residents start performing LC in their PGY-3 (post-graduate year 3) under the supervision of a senior surgeon.

The supervision was pure purely observational as the senior surgeon did not scrub nor assist in the surgery. The patients’ data were coded and entered into an Excel file. After validation, statistical analysis was performed using the analysis software Epi Info following three steps:

· Step 1: We performed a descriptive analysis of data collected. The results were presented as percentage and as mean ± standard deviation.

· Step 2: Univariate analysis was performed for comparing averages and percentages using statistical tests such as Student's t-test, chi-square test, and Fisher's test.

· Step 3: Multivariate analysis was performed using logistic stepping-down regression.

The results are reported in graphs and tables. A p-value of <0.05 was considered significant. The means and standard deviations of continuous variables were calculated and expressed, while the 𝜒2 test was used to express dichotomous variables. Student’s t-test was used to analyze continuous variables.

## Results

Our study included 433 patients; 34% of procedures were performed by senior surgeons and 66% were performed by resident trainees. The age of patients ranged from 18 to 87 years. The mean age was 49 years, with a female predominance (sex ratio=1:6); 30% of patients were suffering from associated comorbidities. All patients had undergone abdominal ultrasound, and only 18% had undergone a CT scan. Demographic outcomes are summarized in Table 1.

**Table 1:** Demographics

	Senior surgeon (n=146)	Resident (n=287)	P-value
Mean age	49.7	49.4	0.829
Sex ratio	1:6	1:6	0.885
Comorbidity	45 (31%)	88 (30%)	0.528
Surgical backgrounds	21 (14%)	24 (8%)	0.203
Duration of symptoms (in months)	5.6	6	0.462

Among the 433 LCs performed, 54 were reported comprising intra-operative difficulties. Dense adhesion, inflammatory, and anatomic causes were the most common difficulties faced by the surgeons. The release of adherence and Calot’s triangle dissection require massive diligence in order to avoid biliary duct, vascular, and intestinal injuries. The number of intra-operative difficulties was 32 (11%) in residents’ sample and 22 (15%) in senior surgeons’ sample (p=0.509) (Table 2).

**Table 2:** Outcomes

	Senior surgeons (n=146)	Residents (n=287)	P-value
Intra-operative difficulties	22 (15%)	32 (11%)	0.156
Intra-operative complications	3 (2%)	11 (3.8%)	0.368
Conversion	12 (8%)	23 (8%)	0.538
Drainage	93 (63%)	199 (69%)	0.141
Operative time (in minutes)	61	96	<0.001
Mean hospital stay (in days)	3.09	3.95	<0.001
Post-operative complications	2 (1.3%)	9 (3.1%)	0.223
Return to the operating room	0	3 (1%)	0.290
Mortality	0	0	-

**Figure 2:**
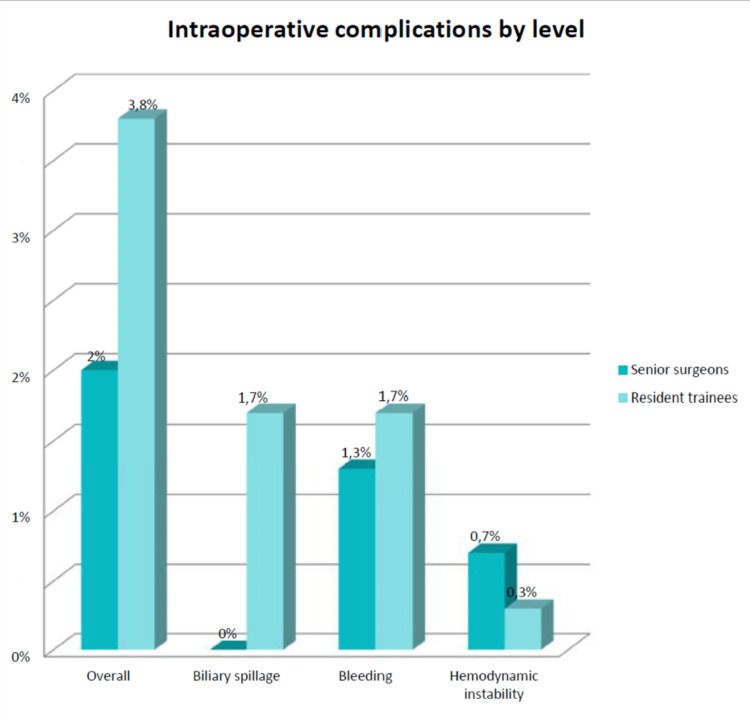
Intra-operative complications according to the surgeon’s level

Intra-operative complications included biliary spillage, bleeding, and hemodynamic instability. We notice that no case of intestinal injury was reported. The number of intra-operative complications was 11 (4%) in residents and three (2%) in senior surgeons’ sample (2%) p=0.368 (Figure 2).

**Figure 3:**
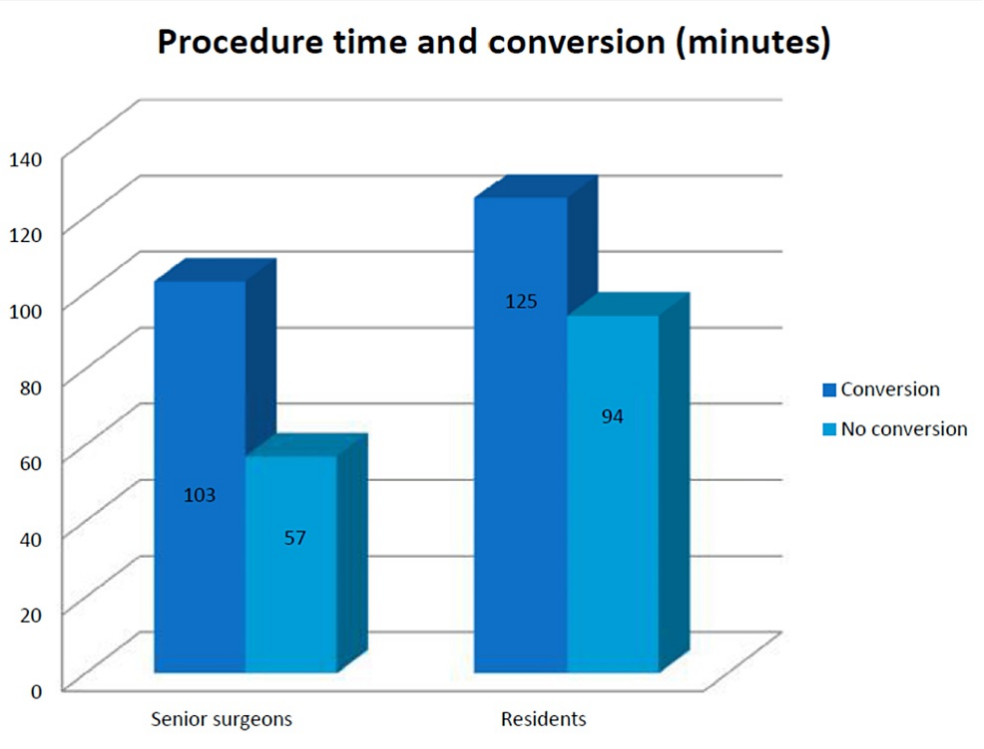
Procedure duration and conversion rate

The majority of cases where laparoscopy has been converted to laparotomy are due to difficulties with identifying anatomy and the inability to safely continue with the laparoscopic procedure. In terms of conversion, the resident cohort had a percentage of 8% (23/287), while among senior surgeons, it was 8.2% (12/146) (p=0.538). The main causes of conversion were dense adhesion, uncontrolled bleeding, iatrogenic trauma, anatomic variation, and technic difficulties (Figure 3).

A total of 287 LCs were performed by resident trainees. Within the resident group, a total of 62 LC was performed by PGY-3, 117 by PGY-4, and 108 by PGY-5. Intra-operative difficulties included dense adhesions, inflammatory changes, and unclear anatomy.

We noticed no significant difference between the three levels: 11.9% in PGY-3 (p=0.593), 11.9% in PGY-4 (p=0.398), and 9.2% in PGY-5 (p=0.365).

The residents' sample reported 14 instances of intra-operative complications. It was observed that junior resident trainees had a slightly higher incidence of drainage compared to senior residents. Operative time was significantly longer in resident trainees compared to senior surgeons. In residents’ sample, operative time was significantly longer in PGY-3 (Table 3).

**Table 3:** Residents’ outcomes PGY, post-graduate year

	PGY-3 (n=62)	PGY-4 (n=117)	PGY-5 (n=108)	P-value
Intra-operative complications	2 (3.2%)	4 (3.4%)	5 (4.5%)	0.586
Conversion	5 (8%)	11 (9.4%)	7 (6.5%)	0.723
Drainage	46 (74%)	80 (68%)	73 (67%)	0.640
Operative time (in minutes)	103	93	92	0.066
Mean hospital stay (in days)	4.2	3.6	4.2	0.017
Post-operative complications	2 (3.2%)	4 (3.4%)	5 (4.6%)	0.603
Return to the operating room	1 (1.6%)	2 (1.7%)	0	0.400
Mortality	0	0	0	-

## Discussion

To our knowledge, this is the first study specifically examining the impact of resident-led and resident-performed procedures on operative times and outcomes of LC in Morocco.

At present, LC is one of the most common surgical operations performed by surgical residents in the United States, with evidence of increased rates during the last few years. This operation is considered to be very effective and safe in the hands of American trainees, who perform more than 100 laparoscopies during their residency, with 68% of these consisting of cholecystectomies [3].

In Morocco, surgical residents receive the major part of their educational program at the University Hospital, which is a third-level hospital, with a low LC rate. Furthermore, the safety restrictions and the lack of hospitalization beds and medical staff negatively affect the productivity of general surgical trainees, with a consequent decrease in operative volume and autonomy.

The mean age in our series was 49 years, with extremes ranging from 18 to 87 years. There was no significant difference between senior surgeons’ group and residents’ group (p=0.829). It has been observed that in our series, the advanced age does not contraindicate laparoscopic surgery; the extreme age was 87 years. Different studies demonstrated that hormones are a non-negligible cause for gallstones formation [4,5]. In our series, 86% of patients were female with a sex ratio of 1:6.

As anticipated, our findings indicate that LCs carried out by residents took significantly longer than those performed by staff surgeons, with an average duration of 96 minutes compared to 61 minutes (p<0.001). Böckler et al. also reported a similar discrepancy, although with longer durations than what we observed; compared to senior surgeons, residents took an average of 119 minutes to complete the procedure, while senior surgeons completed it in 97 minutes on average [6]. The variation in the length of the operation can be attributed, in part, to the lower surgical proficiency of the resident, as supported by previous literature. Additionally, it may also be due to the staff surgeon's approach of teaching the resident that time should not be prioritized and that they should remain completely focused even during seemingly straightforward procedural steps [7,8]. According to our study, we noticed that over the course of five years of general surgery training, there is a gradual improvement in the learning curve for LC, resulting in a decrease in the procedure's duration, with durations of 103 minutes in PGY-3, 93 minutes in PGY-4, and 92 minutes in PGY-5. Kauvar et al. have reported a comparable outcome in their study, where the average duration of LC carried out by residents during the first three years of training was 88 minutes, in contrast to 73 minutes in the last two years of training [7,8].

Compared to European and American recent studies, the operative time in procedures performed by our residents is still quite long. Actually, in most of these countries, LC is performed by residents since their PGY-2 [3,8-10].

To ensure safety, surgeries with a higher likelihood of complications are entrusted to senior surgeons. Consequently, the rate of intra-operative difficulties was comparatively higher in senior surgeons at 15%, as opposed to 11% in residents, but the difference between the two groups was not statistically significant (p=0.156). Traditionally, a cholecystectomy involved the routine insertion of a sub-hepatic drain to minimize the risk of intra-abdominal abscesses, post-surgical bleeding, and biliary fistulas. However, numerous studies have shown that the routine use of a drain does not provide any advantages. Therefore, depending on the surgeon's experience and the patient's circumstances (such as bleeding, signs of gallbladder inflammation, incidental opening, or suspected bile leak), the insertion of a drain may be beneficial [11,12]. Due to the careful implementation of drainage techniques, only one case of post-operative biloma was reported within 30 days after surgery, which is less than 1%.

The overall conversion rate was 8.1% (35/433). The rates of conversion did not differ significantly between operations performed by residents and staff surgeons (8% versus 8.2%). This finding is consistent with the results reported in the majority of studies in which the percentage of conversion varies from 2% to 15% [3,8-11]. It is worth noting that the conversion rates were similar and not statistically significant (p = 0.623) for PGY-3, PGY-4, and PGY-5, with rates of 8%, 9.4%, and 6.5%, respectively. This is in contrast to the findings of Kauvar et al. [7], where the researchers found that residents in their first three years of training had a markedly higher rate of conversion to laparotomy compared to those in their later years of training (8.4% versus 3.7%). The results of Kauvar et al.'s study were attributed to a lack of supervision by senior surgeons for senior trainees and patient selection.

The safety and efficiency of performing LC can be predicted by a surgeon's experience. Intra-operative and post-operative complications can be used as an effective means to evaluate this parameter [13]. In this study, complications such as biliary spillage, bleeding, common bile duct injury, parietal complications, and jaundice were rare and occurred at a similar rate in both groups, with no statistically significant differences (Table 4).

**Table 4:** Comparison of complications rate

Series	Year	Sample size	Complication rate in senior surgeons	Complication rate in residents	P-value
Pariani et al. [8]	2014	569	66	84	0,003
Suuronen et al. [9]	2010	787	55	80	<0.001
Kauvar et al. [7]	2006	562	67	88	<0.05
Bencini et al. [10]	2008	342	50	67	<0.001
Koulas et al. [11]	2006	1370	49	57	0.12
Our series	2020	433	61	96	<0.001

There was a significant difference in the length of hospital stay between the residents' and senior surgeons' sample, with the former having a mean stay of 3.09 days compared to the latter's 3.95 days (p<0.001). This was due to the higher rate of complications and drainage in the residents' sample, which necessitated close and thorough surveillance, resulting in an average of one day more in the hospital. The overall mean hospital stay of 3.66 days was similar to previous studies by Pariani et al. [8] (3.4 days) and Bencini et al. [10] (3 days).

Despite the great number of patients who underwent LC, we did not record any case of post-operative death. Nevertheless, our results can be explained by the exclusion of urgent procedures and the fact that most patients were ASA grade I/II. This finding is comparable to those reported in the literature where studies show a mortality rate of 0% to 0.6% [1]. The extremely low rate of mortality and morbidity reveal the worthwhile implication of the whole medical staff to ensure an adequate management of difficult cases and providing decent medical health care.

During our research, we encountered various challenges that made our work more daunting. One of these difficulties was related to the tracking of post-operative complications in patients who had undergone surgery before the implementation of the computerized data collection system. This was particularly sensitive because there was limited information available in the paper form files about subsequent hospitalizations, which could have resulted in the omission of some delayed complications. Patients' selection might come into account when it comes to intra-operative difficulties and post-operative complications as we have only selected non-urgent procedures. Additionally, a larger sample size comprising a greater number of patients would have been beneficial. This is due to the fact that our hospital, which is a level III facility, treats relatively few cases of uncomplicated gallstones disease.

## Conclusions

Our study has demonstrated that LC can be safely performed by junior surgery trainees even in resource-limited settings. We found that when surgical residents are adequately supervised, appropriately trained, and operate on carefully selected patients, their LCs are just as safe as those performed by senior surgeons. Our structured training program has been successful in enabling junior surgeons to learn the procedure without increasing the risk of serious complications. However, we believe that the implementation of virtual reality training programs could further enhance the skills of surgical trainees. By providing a realistic simulation of the surgery, trainees can develop their skills in a safe and controlled environment, making them better prepared for real-life surgeries. This could reduce unexpected complications and improve the overall safety of LC procedures, even in low-resource settings.
